# Dermatologists’ and Rheumatologists’ Adherence to the Latest Recommendations for Screening of Hydroxychloroquine Retinopathy in Saudi Arabia: A Cross-Sectional Study

**DOI:** 10.7759/cureus.56179

**Published:** 2024-03-14

**Authors:** Asail S Alghamdi, Ghaida B AlQefari, Khalil Alduraibi, Maryam Al-Amer, Basil A Alharbi, Ahmed N Alqefari, Hana N Alqifari, Meshal M Alhameedy

**Affiliations:** 1 College of Medicine, Albaha University, Al Baha, SAU; 2 Medicine, Qassim University, Buraydah, SAU; 3 General Medicine, College of Medicine and Surgery, King Saud University, Riyadh, SAU; 4 Medicine, College of Medicine, Jazan University, Jazan, SAU; 5 General Surgery, Qassim University, Buraydah, SAU; 6 College of Medicine and Surgery, Qassim University, Buraydah, SAU; 7 Statistics and Operations Research, College of Science, Qassim University, Buraydah, SAU; 8 Dermatology, King Fahad Specialist Hospital, Buraydah, SAU

**Keywords:** aao recommendation, screening, adherence, retinopathy, hcq, hydroxychloroquine

## Abstract

Introduction: Hydroxychloroquine (HCQ) is used to manage the symptoms of inflammatory rheumatic and dermatologic disorders. However, HCQ retinopathy is a serious side effect because even after the drug is discontinued, irreversible vision loss may occur and may continue to progress. According to the American Academy of Ophthalmology (AAO), the recent recommendation for HCQ dosing is ≤5 mg/kg of real body weight, with baseline ophthalmologic screening during the first year of HCQ treatment and yearly screening after five years of continuous use of HCQ medication, unless the patient is at high risk or symptoms have developed. Nonetheless, this study aims to assess dermatologists’ and rheumatologists’ adherence in Saudi Arabia to the 2016 AAO HCQ retinal toxicity guidelines.

Methods: A questionnaire-based cross-sectional study was conducted on dermatologists and rheumatologists in Saudi Arabia. It was conducted between August and September 2022 and questionnaires were sent to dermatologists and rheumatologists via their professional emails or accounts.

Results: The collected sample consisted of 635 participants; males and females represented 54% and 46%, respectively; 47.6% were consultants; 50.1% were rheumatologists; and 49.9% were dermatologists. Approximately 28.2% of the participants reported treating one to three patients with HCQ in the previous year. Only 45.4% of the respondents stated that the ideal recommended HCQ dose for reducing the risk of retinopathy is "≤ 5 mg/kg of the real body weight." More than 50% of the respondents stated that systemic lupus erythematosus was the most common disease for which they used HCQ. Additionally, 36.5% of the physicians screened patients during the first year of HCQ treatment. We found significant associations between practice levels and specialty practice-related questions with a p-value of less than 0.05, except for the specialty practice-related question, "What is the most common disease for which you use HCQ?" with a p-value of 0.074. Also, we found significant associations between all demographic variables and screening-related variables with a p-value of less than 0.05, with two exceptions: no significant associations were found between specialty area and the screening-related question, "Do you recommend screening tests for all patients starting treatment with HCQ?" at p = 0.270, and gender and the screening-related question, "When would you recommend screening tests for a patient without risk?" at p = 0.142.

Conclusions: Dermatologists and rheumatologists in Saudi Arabia have shown poor adherence to the most recent AAO recommendations. Educating physicians and patients about the AAO guidelines is needed for HCQ to be used in a way that is both effective and safe.

## Introduction

Hydroxychloroquine (HCQ) is an antimalarial medication commonly prescribed in the fields of rheumatology and dermatology. However, its administration can lead to various adverse effects [1-3]. HCQ-induced retinal toxicity is a rare but serious side effect. Even after discontinuation of the medication, irreversible vision loss may occur and may continue to progress [4]. The retinal toxicity associated with HCQ is typically characterized by the manifestation of a "bull's-eye" retinopathy, which is characterized by parafoveal atrophy of the outer retina and retinal pigment epithelium [5]. A recent study was conducted on a sample of 3325 patients who had been consistently using HCQ for a minimum of five years [6]. The study revealed that the overall cumulative incidences were 2.5% and 8.6% at 10 and 15 years, respectively [6]. The study reported that the incidence rates of retinopathy after 15 years were 21.6%, 11.4%, and 2.7% for doses higher than 6 mg/kg per day, doses between 5 and 6 mg/kg per day, and doses of 5 mg/kg per day or lower, respectively [6]. In another study published in 2022, 910 patients underwent screening for retinopathy over a two-year period [7]. Among this group, 566 individuals were identified as being at risk for retinopathy [7]. The findings of this study revealed that the overall prevalence of HCQ retinopathy was 1.09% among all patients who underwent screening, while the prevalence was slightly higher at 1.76% among those identified as being at risk [7]. Before 2014, physicians thought that the risk of HCQ retinopathy was low. However, over the past 10 years, improvements in modern, highly sensitive screening methods have made it possible to spot the first signs of HCQ retinopathy [8]. As a result, the number of patients diagnosed with HCQ retinopathy has increased. These advanced modalities include multifocal electroretinography (mfERG), fundus autofluorescence, and spectral-domain optical coherence tomography (SD-OCT) with automated 10-2 visual field assessment (VFA) [8]. The American Academy of Ophthalmology's (AAO) most recent recommendations recommended baseline ophthalmologic screening during the first year of HCQ use and yearly screening after five years of continuous HCQ medication use, unless the patient is at high risk or symptoms have appeared [9]. In 2019, the adherence of Saudi Arabian dermatologists to HCQ screening recommendations for retinopathy was investigated among 76 dermatologists. Based on this research, it was found that the current standard of care for HCQ toxicity screening is lacking in several ways, including the use of the right diagnostic tests, the right amount of time between follow-ups, the prompt withdrawal of medications, and the identification of the causative agents [10]. Despite the limitations of this research regarding the sample size, no further studies were done in Saudi Arabia. Moreover, dermatologists were the only participants, while HCQ is used in many different specialties. In rheumatology, HCQ is used as first-line therapy in systemic lupus erythematosus (SLE) [11]. On the other hand, rheumatologists who are treating patients with SLE in the USA are aware of the guidelines and recommendations, and they recognize the importance of collaborating with ophthalmologists to prevent retinal toxicity, according to a study conducted in 2020 to examine rheumatologists' practices regarding HCQ and the AAO 2016 guidelines for monitoring HCQ retinal toxicity [12]. Screening is essential for detecting early retinopathy before lesions become evident on ophthalmoscopy since, at that time, the damage continues to progress even after the drug is stopped and may ultimately compromise central vision [13-15]. Through screening, we may significantly reduce the chance of late progression and central vision loss [16]. Therefore, our study aims to evaluate the extent to which dermatologists and rheumatologists in Saudi Arabia are aware of the latest guidelines for HCQ retinopathy screening and whether they are implementing these guidelines in their clinical practice. By assessing their knowledge and adherence to the guidelines, the study can identify any gaps or challenges in the implementation of the guidelines and suggest ways to improve the quality of care for patients taking HCQ.

## Materials and methods

A cross-sectional study was conducted in Saudi Arabia from August 2022 to October 2022. The study population consisted of dermatologists and rheumatologists from Saudi Arabia. Ethical approval was obtained from the Regional Research Ethics Committee of Qassim Province (registration number: 607-44-7359).

Dermatologists and rheumatologists who work as board-certified consultants and specialists in Saudi Arabia with academic degrees were included. Residents and those who work outside Saudi Arabia were excluded.

A structured questionnaire was used as a study tool. This questionnaire was adapted and validated from previous studies conducted in Saudi Arabia and elsewhere [10,12]. Dermatologists and rheumatologists who agreed to participate in this study and met the inclusion criteria could fill out an anonymous questionnaire. The questionnaire included demographic data, specialty-related questions, screening-related questions, follow-up-related questions, factors perceived by the physician to induce retinal toxicity, and abnormal screening tests. It was an electronic questionnaire designed via Google Forms (Google, Mountain View, CA) that was sent to dermatologists and rheumatologists via their professional email addresses or professional accounts.

The collected data were analyzed and described using descriptive statistics and the Statistical Package for Social Sciences (SPSS) software version 25 (IBM Corp., Armonk, NY). Before entering the data into SPSS, it was cleaned and described using descriptive statistics. Descriptive statistics (frequency and percentage) were used to analyze the data. The comparison of categorical variables such as gender, practice level, and specialty with other variables was performed using chi-square tests to see whether there was any association between demographic variable groups and other variables. The statistical significance level was assigned at p = 0.05.

## Results

The study included 635 physicians who had completed the questionnaire, with 317 dermatologists and 318 rheumatologists. A total of 54% of responses were from males and 46% were from females. There were 47.6% consultants and 52.4% specialists. The demographic characteristics are listed in Table 1.

**Table 1:** Demographic data

Demographic	Number of responses (%)
Gender	
Male	343 (54%)
Female	292 (46%)
Practice level	
Consultant	302 (47.6%)
Specialist	333 (52.4%)
Specialty	
Dermatology	317 (49.9%)
Rheumatology	318 (50.1%)

The questions related to dermatology practice are listed in Table 2. Of the participants, 28.2% reported treating one to three patients with HCQ during the past year, 32.6% reported treating four to six patients, and 19.8% treated more than 10 patients. Of the participants, 28.7% reported prescribing 400 mg per day of HCQ; 28.2% of the participants reported prescribing 5 mg/kg of real body weight (RBW). In addition, 22.4% of the participants prescribed 200 mg once a day (OD)of HCQ, and 16.2% prescribed 6.5 mg/kg of HCQ. Of them, 45.4% reported that the ideal recommended dose of HCQ to minimize retinopathy risk is "equal to or less than 5 mg/kg of the RBW," and 22.7% of them reported 200 mg once daily. In addition, 33.4% stated prescribing HCQ between three and four years, while 25.4% stated prescribing HCQ for more than four years. There were 358 (56.4%) participants who stated that SLE was the most common disease for which they used HCQ. A total of 85.5% measured RBW at regular intervals. Of the participants, 43.3% recommended five to 10 years as the maximum duration of treatment.

**Table 2:** Dermatology practice-related questions OD: once a day; BID: twice a day; HCQ: hydroxychloroquine. ** Multiple response question.

Variables	Number of responses (%)
In the past year, how many patients did you care for, who were treated with HCQ?	
1-3	179 (28.2%)
4-6	207 (32.6%)
7-10	123 (19.4%)
More than 10	126 (19.8%)
What dose of HCQ do you usually prescribe?	
200 mg OD	142 (22.4%)
200 mg BID	182 (28.7%)
6.5 mg/kg	103 (16.2%)
5 mg/kg real body weight	179 (28.2%)
5 mg/kg ideal body weight	29 (4.6%)
What is the optimal recommended dose for HCQ to reduce the risk of retinopathy?	
200 mg OD (once daily)	144 (22.7%)
200 mg BID (twice daily)	133 (20.9%)
Equal to or less than 5 mg/kg of the real body weight (RBW)	288 (45.4%)
Equal to or less than 5 mg/kg of the ideal body weight (IBW)	70 (11.0%)
What is the average time your patients are currently treated with HCQ?	
1-2 years	127 (20.0%)
3-4 years	212 (33.4%)
Less than 1 year	135 (21.3%)
More than 4 years	161 (25.4%)
What is the most common disease for which you use HCQ?**	
Systemic lupus erythematosus	358 (56.4%)
Discoid lupus erythematosus	111 (17.5%)
Dermatomyositis	123 (19.4%)
Lichen planus	27 (4.3%)
Morphea	10 (1.6%)
Other	6 (0.9%)
Do you measure body weight at regular intervals?	
Yes	543 (85.5%)
No	92 (14.5%)
What is the maximum duration of treatment recommended?	
<5 years	164 (25.8%)
5-10	275 (43.3%)
10-20	120 (18.9%)
>20 years	76 (12.0%)

The participants who recommended screening all patients starting HCQ treatment were 54.3%, while another 40% said they sometimes recommended screening. In addition, 35.4% of participants performed the screening test before starting HCQ treatment, and 36.5% stated that they screened for patients in the first year of HCQ treatment. The primary tests that participants stated that they recommended were up to the ophthalmologist (26.6%), visual field testing (48.7%), ocular examination (45.9%), and spectral-domain optical coherence tomography (46.7%). Regarding patients with no risk factors, 30.7% of participants stated screening them before initiating HCQ treatment, and 35.7% recommended screening patients without risk factors during the first year of HCQ treatment (Table 3).

**Table 3:** Screening-related questions HCQ: hydroxychloroquine. ** Multiple response question.

Variables (N = 635)	Number of responses (%)
Do you recommend screening tests for all patients starting treatment with HCQ?	
Yes	345 (54.3%)
Sometimes	254 (40.0%)
No	36 (5.7%)
When do you perform the screening tests?	
Before initiating HCQ treatment	225 (35.4%)
During the first year of HCQ treatment	232 (36.5%)
During the first 5 years of HCQ treatment	77 (12.1%)
Only in patients at risk	101 (15.9%)
Which tests would you recommend for screening?^**^	% of cases
Ocular examination	45.9%
Color testing	35.8%
Visual field testing	48.7%
Spectral-domain optical coherence tomography	46.7%
Up to the ophthalmologist	26.6%
When would you recommend screening tests for a patient without risk?	
Before initiating HCQ treatment	295 (30.7%)
During the first year of HCQ treatment	227 (35.7%)
During the first 5 years of HCQ treatment	154 (24.3%)
Only in patients at risk	50 (9.3%)

In terms of the recommended time for follow-up screening tests, 39.8% stated "yearly after five years of treatment," 37.8% stated "yearly after five years of treatment" for patients with no risk, while (81.3%) stated "yearly within five years of treatment" for patients at risk (Table 4).

**Table 4:** Follow-up-related questions HCQ: hydroxychloroquine.

Follow-up-related questions	Number of responses (%)
What is the recommended time for follow-up screening tests for patients without risk?	
Yearly, after 3 years of treatment	240 (37.8%)
Yearly, after 5 years of treatment	253 (39.8%)
Yearly, after starting the treatment	142 (22.4%)
What is the recommended time for follow-up screening tests for patients at risk?	
Yearly, within 5 years of treatment	516 (81.3%)
Yearly, after 5 years of treatment	119 (18.7%)
Have you ever stopped HCQ therapy because of an abnormal screening test?	
No	182 (28.7%)
Yes	453 (71.3%)
If one of the screening tests is abnormal, what would be your next step?	
Decrease the dose	175 (27.6%)
Follow ophthalmology recommendation	248 (39.1%)
Stop the medication	212 (33.4%)

The major risk factors for retinal toxicity that participants stated were HCQ treatment duration (47.2%), HCQ dose (46.8%), renal disease (43.3%), and macular disease (37.0%) (Table 5).

**Table 5:** Factors considered as risk factors for retinal toxicity HCQ: hydroxychloroquine. ** Multiple response question.

What are the major risk factors for retinal toxicity?^**^	% of cases
Age > 70	34.8%
Age < 30	14.2%
HCQ treatment duration	47.2%
HCQ dose	46.8%
Cumulative HCQ dose	35.3%
Macular disease	37.0%
Concomitant tamoxifen use	26.8%
Renal disease	43.3%
Liver disease	25.5%
Genetic	9.3%

**Figure 1:**
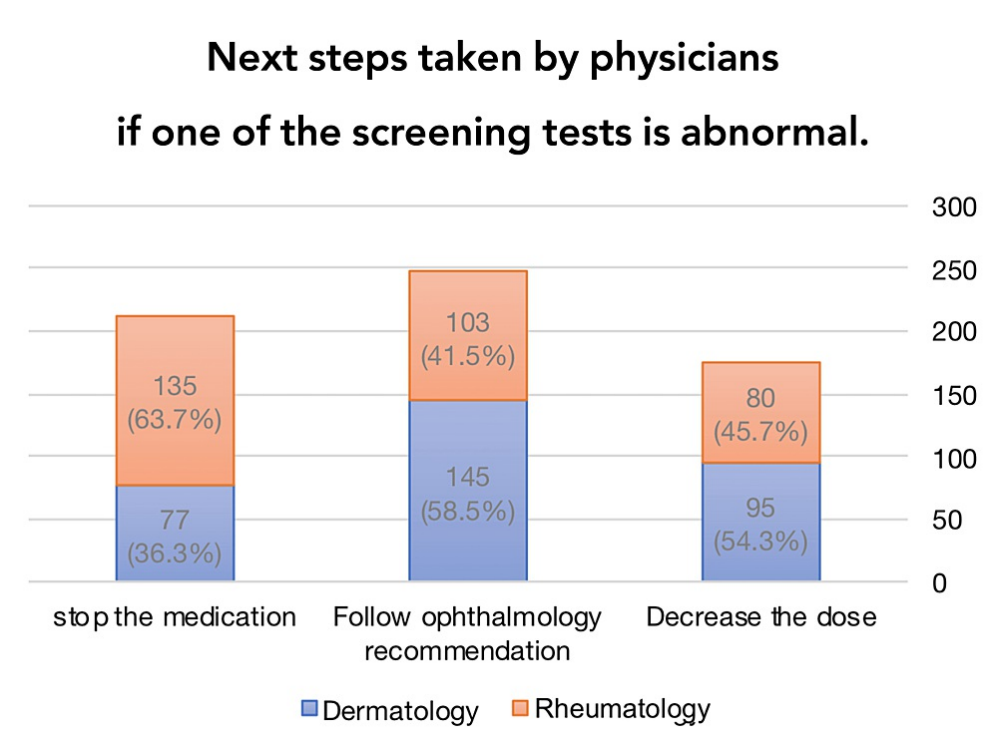
Next steps taken by physicians if one of the screening tests is abnormal The next steps taken by dermatology and rheumatology physicians after an abnormal result in one of the screening tests are illustrated in percentages.

In response to the question, "If one of the screening tests is abnormal, what would be your next step?" Most participants replied, "Follow ophthalmology recommendations" (n = 248, 39.1%; where 58.5% were dermatologists and 41.5% were rheumatologists), followed by "Stop the medication" (n = 212; where 36.3% were dermatologists and 63.7% were rheumatologists) and "Decrease the dose" (n = 175, 27.6%; where 54.3% were dermatologists and 45.7% were rheumatologists) (Figure 1).

**Figure 2:**
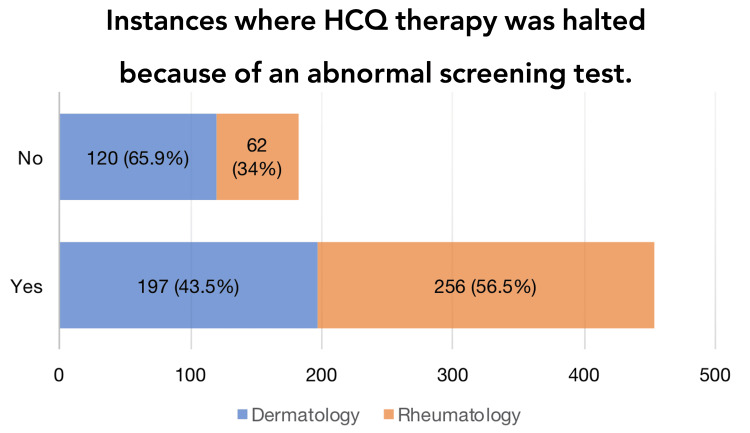
Instances where HCQ therapy was halted because of an abnormal screening test Instances where dermatology and rheumatology physicians stopped HCQ therapy due to an abnormal screening test are shown as percentages. HCQ: hydroxychloroquine.

Most participants (n = 453, 71.3%) stated that they had discontinued treatment because of abnormal screening results (Figure 2).

We found significant associations between practice levels and dermatology practice-related questions with a p-value less than 0.05, except for the dermatology practice-related question, "What is the most common disease for which you use HCQ?" with a p-value of 0.074. In terms of gender, there were significant relationships between gender and dermatology practice-related questions, with the exception of "Do you measure body weight at regular intervals?" and "What maximum duration of treatment is recommended?" with p-values of 0.330 and 0.050, respectively. Regarding specialty area, there were significant relationships between specialty area and dermatology practice-related questions, with the exception of "What is the optimal recommended dose for HCQ to reduce the risk of retinopathy?" and "What is the most common disease for which you use HCQ?" with p-values of 0.156 and 0.102, respectively (Table 6).

**Table 6:** Chi-square comparison of dermatology practice-related questions to participant demographics OD: once a day; BID: twice a day; HCQ: hydroxychloroquine; SLE: systemic lupus erythematosus; DLE: discoid lupus erythematosus. * Significant result.

Comparison	Practice level			Gender			Specialty		
	Consultant	Specialist	p	Female	Male	p	Dermatology	Rheumatology	p
In the past year, how many patients did you care for who were treated with HCQ?									
01-Mar	73 (40.8%)	106 (59.2%)	0.000*	97 (54.2%)	82 (45.8%)	0.019*	125 (69.8%)	54 (30.2%)	0.000*
04-Jun	82 (39.6%)	125 (60.4%)		79 (38.2%)	128 (61.8%)		100 (48.3%)	107 (51.7%)	
07-Oct	67 (54.5%)	56 (45.5%)		57 (46.3%)	66 (53.7%)		61 (49.6%)	62 (50.4%)	
More than 10	80 (63.5%)	46 (36.5%)		59 (46.8%)	67 (53.2%)		31 (24.6%)	95 (75.4%)	
What dose of HCQ do you usually prescribe?									
200 mg OD	56 (39.4%)	86 (60.6%)	0.007*	70 (49.3%)	72 (50.7%)	0.001*	94 (66.2%)	48 (33.8%)	0.000*
200 mg BID	94 (51.6%)	88 (48.4%)		75 (41.2%)	107 (58.8%)		107 (58.8%)	75 (41.2%)	
6.5 mg/kg	38 (36.9%)	65 (63.1%)		33 (32.0%)	70 (68.0%)		25 (24.3%)	78 (75.7%)	
5 mg/kg real body weight	97 (54.2%)	82 (45.8%)		95 (53.1%)	84 (46.9%)		72 (40.2%)	107 (59.8%)	
5 mg/kg ideal body weight	17 (58.6%)	12 (41.4%)		19 (65.5%)	10 (14.5%)		19 (65.5%)	10 (34.5%)	
What is the optimal recommended dose for HCQ to reduce the risk of retinopathy?									
200 mg OD	83 (57.6%)	61 (42.4%)	0.016*	57 (39.6%)	87 (60.4%)	0.000*	80 (55.6%)	64 (44.4%)	0.156
200 mg BID	54 (40.6%)	79 (59.4%)		44 (33.1%)	89 (66.9%)		57 (42.9%)	76 (57.1%)	
5 mg/kg of the real body weight	128 (44.4%)	160 (55.6%)		158 (54.9%)	130 (45.1%)		148 (51.4%)	140 (48.6%)	
5 mg/kg of the ideal body weight	37 (52.9%)	33 (47.1%)		33 (47.1%)	37 (52.9%)		32 (45.7%)	38 (54.3%)	
What is the average time your patients are currently treated with HCQ?									
1-2 years	68 (53.5%)	59 (46.5%)	0.000*	60 (47.2%)	67 (52.8%)	0.007*	89 (70.1%)	38 (29.9%)	0.000*
3-4 years	92 (43.4%)	120 (56.6%)		99 (46.7%)	113 (53.3%)		114 (53.8%)	98 (46.2%)	
Less than 1 year	47 (34.8%)	88 (65.2%)		46 (34.1%)	89 (65.9%)		58 (43.0%)	77 (57.0%)	
More than 4 years	95 (59.0%)	66 (41.0%)		87 (54.0%)	74 (46.0%)		56 (34.8%)	105 (65.2%)	
What is the most common disease for which you use HCQ?									
SLE	170 (47.5%)	188 (52.5%)	0.074	187 (52.2%)	171 (47.8%)	0.000*	172 (48.0%)	186 (52.0%)	0.102
DLE	56 (50.5%)	55 (49.5%)		51 (45.9%)	60 (54.1%)		68 (61.3%)	43 (38.7%)	
Dermatomyositis	50 (40.7%)	73 (59.3%)		33 (26.9%)	90 (73.2%)		58 (47.2%)	65 (52.8%)	
Lichen planus	15 (50.0%)	12 (44.4%)		14 (51.9%)	13 (48.1%)		12 (44.4%)	15 (55.6%)	
Morphea	5 (50%)	5 (50%)		4 (40.0%)	6 (60.0%)		3 (30.0%)	7 (70.0%)	
Other	6 (100%)	0 (0%)		3 (50%)	3 (50%)		4 (66.7%)	2 (33.3%)	
Do you measure body weight at regular intervals?									
Yes	246 (45.3%)	297 (54.7%)	0.006*	254 (46.8%)	289 (53.2%)	0.33	255 (47.0%)	288 (53.0%)	0.000*
No	56 (60.9%)	36 (49.1%)		38 (41.3%)	54 (58.7%)		62 (67.4%)	30 (32.6%)	
What maximum duration of treatment is recommended?									
<5 years	70 (42.7%)	94 (57.3%)	0.000*	78 (47.6%)	86 (52.4%)	0.05	113 (68.9%)	51 (31.1%)	0.000*
05-Oct	118 (42.9%)	157 (57.1%)		112 (40.7%)	163 (59.3%)		135 (49.1%)	140 (50.9%)	
Oct-20	59 (49.2%)	61 (50.8%)		58 (48.3%)	62 (51.7%)		56 (46.7%)	64 (53.3%)	
>20 years	55 (72.4%)	21 (27.6%)		44 (57.9%)	32 (42.1%)		13 (17.1%)	63 (82.9%)	

We found significant associations between all demographic variables and screening-related variables with a p-value less than 0.05, with two exceptions. No significant associations were found between specialty area and the screening-related variable, "Do you recommend screening tests for all patients starting treatment with HCQ?" at p = 0.270, and gender and the screening-related variable, "When would you recommend screening tests for a patient without risk?" at p = 0.142 (Table 7).

**Table 7:** Chi-square comparison of screening-related variables to participant demographics HCQ: hydroxychloroquine. * Significant result.

Comparison	Practice level			Gender			Specialty		
	Consultant	Specialist	p	Female	Male	p	Dermatology	Rheumatology	p
Do you recommend screening tests for all patients starting treatment with HCQ?									
Yes	174 (50.4%)	171 (49.6%)	0.054	176 (51.0%)	169 (49.0%)	0.018*	182 (52.8%)	163 (47.2%)	0.270
Sometimes	107 (42.1%)	147 (57.9%)		100 (39.4%)	154 (60.6%)		117 (46.1%)	137 (53.9%)	
No	21 (58.3%)	15 (41.7%)		16 (44.4%)	20 (55.6%)		18 (50%)	18 (50%)	
When do you perform the screening tests?									
Before initiating HCQ treatment	100 (44.4%)	125 (55.6%)	0.032*	99 (44.0%)	126 (56.0%)	0.000*	135 (60.0%)	90 (40.0%)	0.000*
During the first year of HCQ treatment	118 (50.9%)	114 (49.1%)		132 (56.9%)	100 (43.1%)		114 (49.1%)	118 (50.9%)	
During the first 5 years of HCQ treatment	45 (58.4%)	32 (41.6%)		34 (44.2%)	43 (55.8%)		33 (42.9%)	44 (57.9%)	
Only in patients at risk	39 (38.6%)	62 (61.4%)		27 (26.7%)	74 (73.3%)		35 (34.7%)	66 (65.3%)	
Which tests would you recommend for screening?									
Ocular examination	125 (54.1%)	106 (45.9%)	0.028*	110 (47.6%)	121 (52.4%)	0.011*	118 (51.1%)	113 (48.9%)	0.034*
Color testing	78 (43.3%)	102 (56.7%)		77 (42.8%)	103 (57.2%)		75 (41.7%)	105 (58.3%)	
Visual field testing	113 (46.1%)	132 (53.9%)		127 (51.8%)	118 (48.2%)		127 (51.8%)	118 (48.2%)	
Spectral-domain optical coherence tomography	101 (43.0%)	134 (57.0%)		137 (58.3%)	98 (41.7%)		111 (47.2%)	124 (52.8%)	
Up to the ophthalmologist	51 (38.1%)	83 (61.9%)		59 (44.0%)	75 (56.0%)		79 (59.0%)	55 (41.0%)	
When would you recommend screening tests for a patient without risk?									
Before initiating HCQ treatment	78 (40.0%)	117 (60.0%)	0.001*	93 (47.7%)	102 (52.3%)	0.142	121 (62.1%)	74 (37.9%)	0.000*
During the first year of HCQ treatment	99 (43.6%)	128 (56.4%)		104 (45.8%)	123 (54.2%)		100 (44.1%)	127 (55.9%)	
During the first 5 years of HCQ treatment	90 (58.4%)	64 (41.6%)		76 (49.4%)	78 (50.6%)		62 (40.3%)	92 (59.7%)	
Only in patients at risk	35 (59.3%)	24 (40.7%)		19 (32.2%)	40 (67.8%)		34 (57.6%)	25 (42.2%)	

## Discussion

According to the results and our main objective, the majority of dermatologists and rheumatologists did not follow the most recent AAO screening recommendations for HCQ retinopathy. The principal result showed poor adherence to the recent AAO recommendations for screening HCQ retinopathy among dermatologists and rheumatologists. Most of the participants had treated four or more patients with HCQ in the previous year, emphasizing the widespread use of HCQ by dermatologists and rheumatologists. In practice, only a minority prescribed the recommended dose of 5 mg/kg RBW, and only 45% recommended using 5 mg/kg RBW or less, whereas the majority used non-weight-based dosing, which significantly increased the risk of retinopathy. Approximately 54% of the participants were unfamiliar with the latest 2016 AAO guidelines recommending 5 mg/kg of RBW as the recommended dose [9]. About 25% were treated for more than four years, and the majority were prescribed more than the recommended dose, which increases the risk of retinopathy. The maximum recommended duration of HCQ therapy varied. Approximately 30.9% of participants recommend using HCQ for more than 10 years, which significantly increases the risk of retinopathy. Only 43.3% of participants recommended a treatment duration of five to 10 years. The latest AAO guidelines recommend HCQ retinopathy screening during the first year for all patients, regardless of risk [9]. Our results showed that half of the participants recommended screening and 5.7% did not. The majority of the participants were not adherent to the timing of starting screening for retinopathy, and many thought risk factors determined the need for screening within the first year. During the first year of HCQ therapy for a patient without risk, only 36.5% of the physicians prescribed screening tests. After five years of treatment, recommended follow-up screening tests should be performed annually for patients without risk factors [9]. Of the participants, 29.8% chose the correct recommendation. The following are major risk factors for HCQ retinal toxicity: a daily dosage of HCQ over 5 mg/kg RBW; duration of use exceeding five years, especially after 10 years of continuous use if no additional risk factors are present; renal disorder with subnormal glomerular filtration rate; tamoxifen treatment; and macular disorder [9]. In our study, 46.8% of participants recognized daily dosage of HCQ over 5 mg/kg RBW as a major risk factor, 47.2% recognized HCQ treatment duration, 43.3% of participants recognized renal diseases, 26.8% of participants recognized tamoxifen treatment, and 37% of participants recognized macular diseases as major risk factors. In cases of abnormal screening tests, the guideline recommends obtaining additional testing, and if toxicity is suspected, more frequent and detailed examinations should be performed. Once toxicity is confirmed, the prescribing physician should be notified and HCQ should be discontinued unless it is medically critical (e.g., a potential flare-up of SLE) and the patient has been informed of the visual risk and that the drug clears slowly from the body and therefore visual function may continue to slowly deteriorate even in the case of discounting HCQ [9]. According to our data, if screening test results were abnormal, the majority of dermatologists followed ophthalmology recommendations, while the majority of rheumatologists tended to stop the medication immediately. The recommended method for routine screening included both SD-OCT and automated visual fields. The sensitivity of fields is potentially higher; however, it is subjective, and the reliability of patient responses may vary. On the other hand, SD-OCT is an objective method that exhibits high specificity and generally high sensitivity for detecting levels of damage that may have visual significance [9]. In our study, 48.7% of physicians used visual field testing and 46.7% used SD-OCT as a screening method.

A study done in 2019 in Saudi Arabia on dermatologists showed that more than half of the participants reported treating one to three patients with HCQ during the last year. More than half of the participants reported that they prescribed 400 mg per day of HCQ, and 32% of them knew that the recommended dose is "equal to or less than 5 mg/kg RBW" [10]. However, in our study, the minority used the recommended dose of 5 mg/kg of RBW, and the majority used non-weight-based dosing. These discrepancies could be explained by our larger sample size and the inclusion of rheumatologists. In the same study, 61% of the participants reported that they screen patients before initiating HCQ treatment, and 28% of them reported that they screen patients during the first year of HCQ treatment, while our results showed that 35.4% of participants screen their patients before initiating the treatment and 36.5% screen their patients during the first year of HCQ treatment. Another study done on rheumatologists showed baseline screening tests were recommended by 85% of the participants when starting HCQ therapy. A total of 29% of rheumatologists do not initiate HCQ treatment until the completion of a baseline evaluation [17]. Our study showed that 40% sometimes do the screening and 5.7% do not screen at all. The sample size of our study was larger than that of any previously published research in this area; however, our study was cross-sectional, with the possibility of recall and response bias.

## Conclusions

In conclusion, it was found that dermatologists and rheumatologists in Saudi Arabia have poor adherence to the most recent AAO recommendations regarding the time of screening, follow-up, duration, when to stop treatment, and contributing factors. Additionally, the results showed that participants had inadequate awareness of the recommended dose and measurement of RBW before starting HCQ treatment and a lack of adherence to these guidelines in managing patients. To continue using HCQ, the benefits of treatment must outweigh the associated risks. We recommend raising awareness of AAO guidelines among physicians to improve their adherence. Patient education is also important to achieve effective and safe HCQ treatment.
